# Listeria monocytogenes Exploits Mitochondrial Contact Site and Cristae Organizing System Complex Subunit Mic10 To Promote Mitochondrial Fragmentation and Cellular Infection

**DOI:** 10.1128/mBio.03171-19

**Published:** 2020-02-04

**Authors:** Filipe Carvalho, Anna Spier, Thibault Chaze, Mariette Matondo, Pascale Cossart, Fabrizia Stavru

**Affiliations:** a Unité des Interactions Bactéries-Cellules, Institut Pasteur, Paris, France; b Institut National de la Santé et de la Recherche Médicale (INSERM), U604, Paris, France; c Institut National de la Recherche Agronomique (INRA), USC2020, Paris, France; d Université Paris Diderot, Sorbonne Paris Cité, Paris, France; e Plateforme Protéomique, Unité de Spectrometrie de Masse pour Biologie (UTechS MSBio), Institut Pasteur, Paris, France; f Centre National de la Recherche Scientifique (CNRS), USR 2000, Paris, France; g CNRS, SNC5101, Paris, France; UCLA School of Medicine

**Keywords:** *Listeria monocytogenes*, MICOS complex, Mic10, mitochondrial fission, proteomics

## Abstract

Mitochondrial function adapts to cellular demands and is affected by the ability of the organelle to undergo fusion and fission in response to physiological and nonphysiological cues. We previously showed that infection with the human bacterial pathogen Listeria monocytogenes elicits transient mitochondrial fission and a drop in mitochondrion-dependent energy production through a mechanism requiring the bacterial pore-forming toxin listeriolysin O (LLO). Here, we performed quantitative mitochondrial proteomics to search for host factors involved in L. monocytogenes-induced mitochondrial fission. We found that Mic10, a critical component of the mitochondrial contact site and cristae organizing system (MICOS) complex, is significantly enriched in mitochondria isolated from cells infected with wild-type but not with LLO-deficient L. monocytogenes. Increased mitochondrial Mic10 levels did not correlate with upregulated transcription, suggesting a posttranscriptional mechanism. We then showed that Mic10 is necessary for L. monocytogenes-induced mitochondrial network fragmentation and that it contributes to L. monocytogenes cellular infection independently of MICOS proteins Mic13, Mic26, and Mic27. In conclusion, investigation of L. monocytogenes infection allowed us to uncover a role for Mic10 in mitochondrial fission.

## Abstract (precis)

Pathogenic bacteria can target host cell organelles to take control of key cellular processes and promote their intracellular survival, growth, and persistence. Mitochondria are essential, highly dynamic organelles with pivotal roles in a wide variety of cell functions. Mitochondrial dynamics and function are intimately linked. Our previous research showed that Listeria monocytogenes infection impairs mitochondrial function and triggers fission of the mitochondrial network at an early infection stage, in a process that is independent of the presence of the main mitochondrial fission protein Drp1. Here, we analyzed how mitochondrial proteins change in response to L. monocytogenes infection and found that infection raises the levels of Mic10, a mitochondrial inner membrane protein involved in formation of cristae. We show that Mic10 is important for L. monocytogenes-dependent mitochondrial fission and infection of host cells. Our findings thus offer new insight into the mechanisms used by L. monocytogenes to hijack mitochondria to optimize host infection.

## INTRODUCTION

Mitochondria are among the most important eukaryotic organelles due to their role in several essential cellular processes, such as energy production, biosynthesis of metabolic intermediates, calcium storage and signaling, autophagy, and apoptosis, as well as redox and innate immune signaling (1–3). The overall morphology and cellular distribution of the mitochondrial network are controlled by a succession of fusion and fission events referred to as “mitochondrial dynamics.” This dynamic equilibrium is fundamental to meet cellular energetic and metabolic demands and for responses to stress-inducing conditions (4).

Mitochondrial dynamics are governed by a family of large GTPases with membrane-shaping properties necessary to drive fusion or fission of mitochondria (5, 6). Mitochondrial fusion requires the sequential merging of the outer mitochondrial membrane (OMM) and the inner mitochondrial membrane (IMM) by the action of the OMM-anchored mitofusin 1 (Mfn1) and 2 (Mfn2) proteins and of the IMM-bound optic atrophy 1 (Opa1) protein. Mitochondrial fission is mediated mainly by cytosolic dynamin-related protein 1 (Drp1), with the endoplasmic reticulum (ER), actin, and septins also playing significant roles (6, 7). The importance of mitochondrial dynamics in cell physiology is attested by numerous neuromuscular pathologies associated with genetic defects affecting the expression or activity of proteins involved in mitochondrial fusion and fission (8).

Due to their involvement in essential cellular processes, mitochondria represent attractive targets for viral and bacterial pathogens (9–12). Indeed, many pathogenic bacteria were previously shown to modulate mitochondrial dynamics to create the ideal conditions for intracellular replication, immune evasion, and persistence (11, 13–17). We previously explored how mitochondrial function and dynamics are impacted by infection with Listeria monocytogenes (16, 18), a facultative intracellular bacterial pathogen responsible for listeriosis, a life-threatening disease in immunocompromised individuals (19). We showed that L. monocytogenes causes fragmentation of the host mitochondrial network early in infection. This event requires the bacterial pore-forming toxin listeriolysin O (LLO), which promotes calcium influx into the host cell (16), causing a drop in the mitochondrial membrane potential and triggering Drp1-independent mitochondrial fission (18). L. monocytogenes infection has thus revealed an unconventional mechanism of mitochondrial fission, but the mechanistic details and molecular players involved in modulation of mitochondrial dynamics and function upon L. monocytogenes infection remain unclear.

Here, we set out to increase our understanding of the impact of L. monocytogenes infection on host cell mitochondria and to identify novel factors involved in L. monocytogenes-induced mitochondrial fission by performing a quantitative characterization of the mitochondrial proteome upon infection. We report that L. monocytogenes infection significantly upregulates the mitochondrial levels of Mic10, a core subunit of the mitochondrial contact site and cristae organizing system (MICOS) complex (20). We show that this increase in Mic10 abundance requires LLO and is not correlated with increased transcription. Finally, we demonstrate that Mic10 is necessary for L. monocytogenes-induced mitochondrial fragmentation and contributes to bacterial invasion of host cells.

## RESULTS

### Quantitative proteomic analysis of the human mitochondrial response to L. monocytogenes infection.

**FIG 1:**
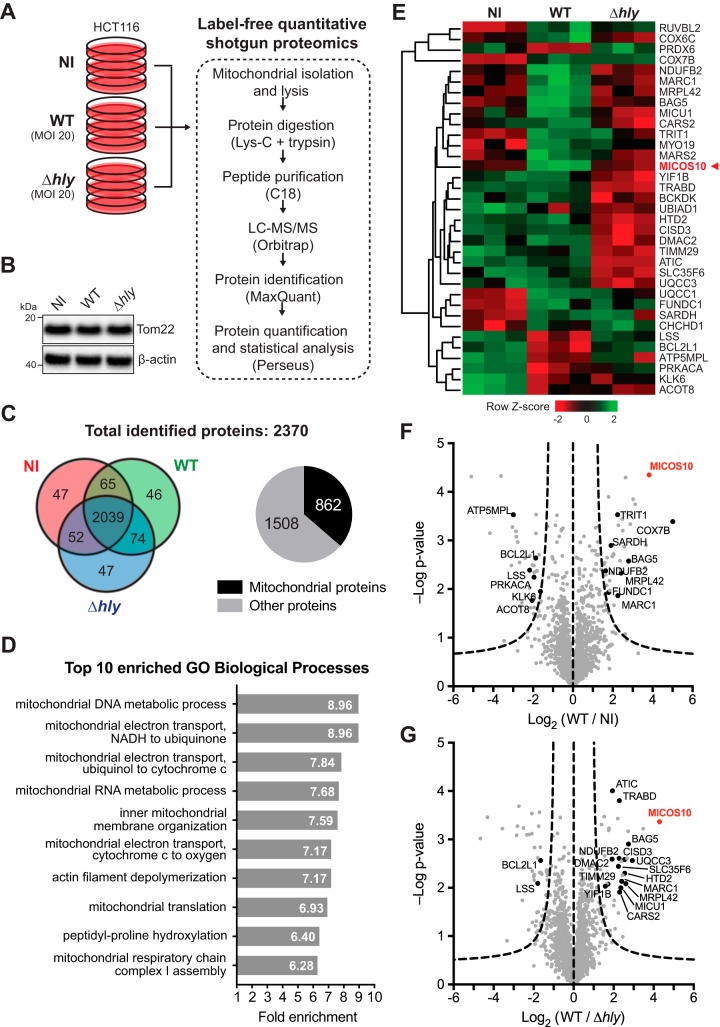
Analysis of changes in the human mitochondrial proteome elicited by L. monocytogenes infection. (A) Schematic diagram of the experimental procedure used for proteomic analysis of human mitochondria isolated from HCT116 cells left uninfected (NI) or infected with wild-type (WT) or LLO-deficient (Δ*hly*) L. monocytogenes. (B) Western blot analysis of whole-cell levels of the mitochondrial protein Tom22 in cell lysates obtained under the conditions mentioned in the panel A legend. This protein was used as the target for the isolation of mitochondria from lysed cells by affinity purification. (C) Venn diagram of the distribution per condition of proteins identified in mitochondria isolated from cells treated as described for panel A. The pie chart shows the distribution of proteins classified as mitochondrial and nonmitochondrial according to MitoMiner’s IMPI database (Q2, 2018). (D) Top 10 most prevalent Gene Ontology (GO) biological processes obtained from functional enrichment analysis (PANTHER gene analysis tool) of all proteins identified in mitochondria isolated from uninfected and L. monocytogenes-infected cells. (E) Heat map diagram showing relative changes in abundance of the 35 proteins annotated as mitochondrial across the different conditions. For each protein, the intensity levels under every set of conditions (each box represents a triplicate) were normalized to the mean value using the Z-score. Intensity levels higher and lower than the mean (black) are indicated in green and red shades, respectively. Proteins are indicated by their gene symbol. (F and G) Volcano plots showing fold change (in log2) in abundance of all identified proteins in mitochondria isolated from cells infected with wild-type (WT) L. monocytogenes compared to uninfected cells (NI) (F) or to cells infected with LLO-deficient L. monocytogenes (Δ*hly*) (G). Triplicates of each condition were analyzed, and a *t* test was performed to determine statistical significance (*y* axis) for each protein. The dashed curves represent the significance limit as determined by Perseus software (FDR = 0.05, S0 = 1) above which changes are deemed statistically significant.

To understand how the human mitochondrial proteome is affected by L. monocytogenes infection, we performed label-free, quantitative proteomic analysis of mitochondria isolated from human cells infected with L. monocytogenes (Fig. 1A). As our cell model, we used the human intestinal epithelial HCT116 cell line, which is rich in mitochondria and efficiently infected by L. monocytogenes. Since L. monocytogenes-induced mitochondrial fission occurs early in infection and requires LLO (16), we performed short-time (2-h) infections with wild-type (WT) L. monocytogenes or with an LLO-deficient (Δ*hly*) mutant strain to focus on LLO-dependent processes. Mitochondria were isolated from infected and noninfected (NI) cell lysates by affinity purification, using a commercially available kit with magnetic bead-coupled antibodies targeting the OMM protein Tom22. We confirmed that *Listeria* infection did not alter the total cellular levels of Tom22 (Fig. 1B) and thus would not affect the efficiency of mitochondrial isolation. Finally, proteins in the mitochondrial extracts were processed for liquid chromatography-tandem mass spectrometry (LC-MS/MS) analysis (Fig. 1A). A total of 2,370 unique proteins were identified, with 2,039 (86%) proteins detected under every set of conditions (Fig. 1C). Among all identified proteins, 862 (36.4%) were annotated as mitochondrial proteins (Fig. 1C), which represents a good degree of mitochondrial enrichment in our samples (compared to the 7% to 8% fraction of mitochondrial proteins in the human proteome [21]) and a high level of coverage of the mitochondrial proteome (53% of 1,626 mitochondrial proteins) annotated in the Integrated Mitochondrial Protein Index (IMPI; version Q2, June 2018), which includes most mitochondrial proteins annotated in MitoCarta (21). This overrepresentation of mitochondrial proteins is reflected in the results of a Gene Ontology (GO) term enrichment analysis, showing 8 mitochondrial terms among the 10 most highly enriched GO biological processes (Fig. 1D).

To identify proteins whose levels are significantly altered upon infection, we performed a statistical test of all identified proteins to select those whose abundance had changed at least 2-fold. We obtained a list of 167 proteins, of which 35 (21%) were annotated as mitochondrial proteins, according to the IMPI (Fig. 1E; see also Table S1 in the supplemental material). Among these are proteins involved in the mitochondrial electron transport chain, such as NADH:ubiquinone oxidoreductase subunit B2 (NDUFB2) and assembly factor DMAC2, ubiquinol-cytochrome *c* reductase assembly factors 1 and 3 (UQCC1 and UQCC3), cytochrome *c* oxidase subunits 6C and 7B (COX6C and COX7B), and F_1_F_O_ ATP synthase subunit 6.8PL (ATP5MPL). Four of these proteins were significantly more abundant in response to infection, suggesting increased activity of the respiratory chain. Other differentially abundant proteins identified in our analysis are associated with mitochondrial protein translation (cysteinyl-tRNA synthetase 2 [CARS2] and methionyl-tRNA synthetase 2 [MARS2], tRNA isopentenyltransferase 1 [TRIT1], and mitochondrial ribosomal proteins CHCHD1 and MRPL42); metabolism of sterols (lanosterol synthase [LSS]), fatty acids (hydroxyacyl-thioester dehydratase type 2 [HTD2]), and branched-chain amino acids (branched-chain ketoacid dehydrogenase kinase [BCKDK]); regulation of mitophagy (Bcl2-associated athanogene 5 [BAG5] [22, 23], FUN14 domain-containing 1 [FUNDC1] [24], and peroxiredoxin 6 [PRDX6] [25]); apoptosis (Bcl2-like protein 1 [BCL2L1]); crista formation (MICOS complex subunit Mic10 [MICOS10]); and organelle transport (myosin 19 [MYO19] [26]). GO enrichment analysis of the 35 differentially abundant mitochondrial proteins did not reveal statistically significantly overrepresented functional pathways. The abundance of 14 (40%) of the 35 mitochondrial proteins was significantly increased upon infection with wild-type L. monocytogenes, whereas it was decreased for 8 proteins (23%) (Table S1). Interestingly, 7 of the 14 upregulated mitochondrial proteins and 4 of the 8 downregulated mitochondrial proteins did not display these changes upon infection with LLO-deficient bacteria (Table S1), indicating that it was LLO triggering such alterations in their abundance.

10.1128/mBio.03171-19.5 TABLE S1 List of the 35 differentially abundant proteins identified by quantitative label-free shotgun proteomics and annotated as “mitochondrial” according to the IMPI database. Proteins with significantly changed levels were determined by performing an ANOVA test with Perseus software (FDR = 0.05, S0 = 1), and pairs of significantly different conditions were determined by Tukey’s *post hoc* test (FDR = 0.05). Proteins are ordered by ANOVA significance level (false-discovery rate [*q* value]). Download Table S1, PDF file, 0.03 MB. Copyright © 2020 Carvalho et al. 2020 Carvalho et al. This content is distributed under the terms of the Creative Commons Attribution 4.0 International license.

We focused our attention on proteins predicted to participate in mitochondrial dynamics or mitochondrial membrane-remodeling processes. Interestingly, we found Mic10 (MICOS10) among the proteins whose mitochondrial levels had increased significantly in response to infection by wild-type but not LLO-deficient L. monocytogenes (Fig. 1E to G). Mic10 is a core subunit of the mitochondrial contact site and cristae organizing system (MICOS) complex, a conserved IMM complex responsible for the formation of crista junctions (the sites where the IMM invaginates to form cristae) (20). Importantly, Mic10 is a small transmembrane protein, whose V-shaped membrane topology and ability to oligomerize confer it membrane-bending properties that are fundamental for driving the formation of crista junctions (27, 28).

### Mic10 is required for L. monocytogenes-induced mitochondrial network fragmentation.

**FIG 2:**
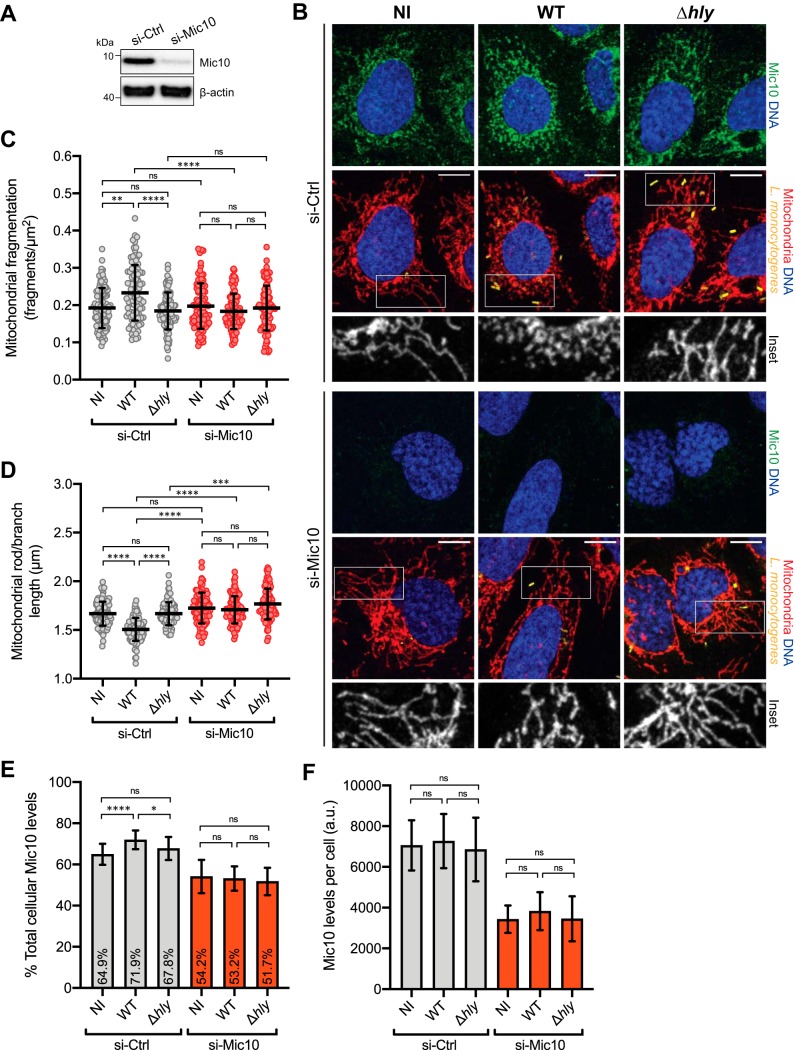
Mic10 knockdown blocks L. monocytogenes-induced mitochondrial fragmentation. (A) Immunoblot analysis of Mic10 levels in U2OS cells transfected with nontargeting control (si-Ctrl) or Mic10-targeting (si-Mic10) siRNAs. Beta-actin protein was used as the loading control. (B) Immunofluorescence analysis of U2OS cells transfected with si-Ctrl or si-Mic10 siRNAs, which were left uninfected (NI) or infected (MOI 50, 2 h) with GFP-expressing wild-type (WT) or LLO-deficient (Δ*hly*) L. monocytogenes. Mic10 proteins are shown in green, mitochondria (anti-Tom20) in red, nuclei (Hoechst 33342) in blue, and bacteria (L. monocytogenes) in yellow. The white box indicates a region of the mitochondrial network magnified (2×) in the inset shown at the bottom (mitochondrial labeling only). Scale bar (top right), 10 μm. (C and D) Quantification of the degree of mitochondrial fragmentation in U2OS cells. Mitochondrial network morphology was analyzed using the morphometric ImageJ plugin tool MiNA on mitochondrion-labeled images. Fragmentation degree per cell was determined by the ratio between the number of individual mitochondrial particles and the mitochondrial area. Scatter plots show mitochondrial fragmentation degree (C) and rod/branch length (D) values for each cell (dots, *n* > 95) and means (horizontal bar) ± standard deviations (SD) and are representative of results from three independent experiments. Significance was determined by a Kruskal-Wallis test (ns, not significant; ***, *P < *0.001; ****, *P < *0.0001). (E and F) Quantification of the mitochondrial fraction (E) and total cellular levels (F) of Mic10 in U2OS cells analyzed as described for panel B. Fluorescence intensities in the entire cell and in mitochondria were measured in Mic10-labeled images using Fiji. Results are expressed as means ± SD (*n* > 95 cells per condition). Significance was determined by a Kruskal-Wallis test (ns, not significant; *, *P < *0.05; ****, *P < *0.0001).

To investigate the role of Mic10 in L. monocytogenes-induced mitochondrial fission, we manipulated Mic10 levels (by small interfering RNA [siRNA]-mediated knockdown or plasmid-mediated overexpression) in host cells before infection and analyzed how the mitochondrial network morphology was affected. We used U2OS cells to assess the effect of Mic10 depletion because their mitochondrial morphology is better suited for microscopy analysis than that of HCT116 cells. U2OS cells were transfected with nontargeting control siRNAs (si-Ctrl) or Mic10-targeting siRNAs (si-Mic10) (Fig. 2A), after which they were left uninfected or infected with wild-type or LLO-deficient bacteria. Cells were fixed and mitochondria immunolabeled for confocal microscopy analysis. In agreement with our previous results, si-Ctrl cells showed a typically tubular mitochondrial network that fragmented upon infection with wild-type but not LLO-deficient bacteria (Fig. 2B). The mitochondrial morphology of si-Mic10 cells was similar to that of si-Ctrl cells, indicating that Mic10 knockdown does not visibly affect mitochondrial network shape. However, unlike the results seen with si-Ctrl cells, increased mitochondrial fragmentation was not detected in si-Mic10 cells in response to wild-type L. monocytogenes (Fig. 2B). To obtain an unbiased, quantitative representation of these observations, we used a semiautomated morphometric tool to analyze the mitochondrial network morphology of a large number of cells, which allowed us to determine the degree of mitochondrial fragmentation per cell. The higher number of fragments per mitochondrial area (Fig. 2C) and the shorter mitochondrial rods and branches (Fig. 2D) confirm the increased mitochondrial fragmentation in si-Ctrl cells infected with wild-type L. monocytogenes compared to uninfected cells or cells infected with LLO-deficient bacteria. In contrast, these parameters were found to have not significantly changed in comparisons of uninfected si-Mic10 cells to si-Mic10 cells infected with wild-type or LLO-deficient L. monocytogenes (Fig. 2C and D). To validate the findings of our proteomic analysis, we employed immunofluorescence to determine if the mitochondrial levels of Mic10 increased upon infection with wild-type L. monocytogenes. Consistent with our proteomic data, infection of si-Ctrl cells with wild-type bacteria led to a small but significant increase in the fraction of mitochondrion-localized Mic10 (Fig. 2E). Interestingly, this occurred without a significant change in the total cellular Mic10 levels (Fig. 2F). We made similar observations in HCT116 cells (see Fig. S1 in the supplemental material), further corroborating the role of Mic10 in mediating mitochondrial fission in response to L. monocytogenes infection.

10.1128/mBio.03171-19.1 FIG S1 Confirmation of Mic10-dependent L. monocytogenes-induced mitochondrial fragmentation in HCT116 cells. (A) Immunoblot analysis of Mic10 levels in HCT116 cells transfected with nontargeting control (si-Ctrl) or Mic10-targeting (si-Mic10) siRNAs. Beta-actin protein was used as the loading control. (B) Immunofluorescence analysis of HCT116 cells transfected with si-Ctrl or si-Mic10 siRNAs, which were left uninfected (NI) or infected (MOI 20, 2 h) with GFP-expressing wild-type (WT) or LLO-deficient (Δ*hly*) L. monocytogenes. Mic10 proteins are shown in green, mitochondria (anti-Tom20) in red, nuclei (Hoechst 33342) in blue, and bacteria (L. monocytogenes) in yellow. The white box indicates a region of the mitochondrial network magnified (2×) in the inset shown at the bottom (mitochondrial labeling only). Scale bar (top right), 10 μm. (C and D) Quantification of the degree of mitochondrial fragmentation in HCT116 cells analyzed as described for panel B. Mitochondrial network morphology was analyzed using the morphometric ImageJ plugin tool MiNA on mitochondrion-labeled images. Fragmentation degree per cell was determined by the ratio between the number of individual mitochondrial particles and the total mitochondrial area. Scatter plots show mitochondrial fragmentation degree (C) and rod/branch length (D) values for each cell (dots, *n* > 50) with means (horizontal bar) ± standard deviations (SD) and are representative of results from three independent experiments. Significance was determined by a Kruskal-Wallis test (ns, not significant; *, *P < *0.05; **, *P < *0.01; ***, *P < *0.001; ****, *P < *0.0001). (E and F) Quantification of the mitochondrial fraction (E) and total cellular levels (F) of Mic10 in HCT116 cells analyzed as described for panel B. Fluorescence intensities in the entire cell and in mitochondria were measured in Mic10-labeled images using Fiji. Results are expressed as means ± SD (*n* > 50 cells per condition). Significance was determined by a Kruskal-Wallis test (ns, not significant; ***, *P < *0.001). Download FIG S1, PDF file, 2.5 MB. Copyright © 2020 Carvalho et al. 2020 Carvalho et al. This content is distributed under the terms of the Creative Commons Attribution 4.0 International license.

**FIG 3:**
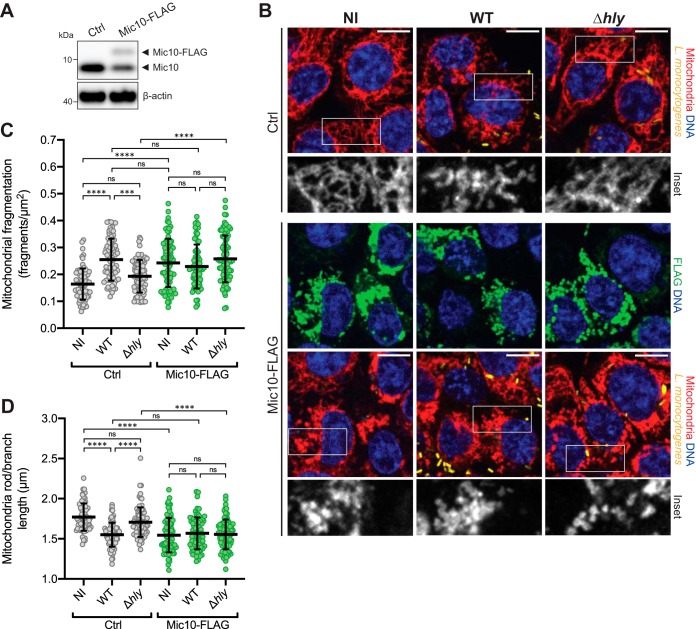
Mic10 overexpression triggers mitochondrial fragmentation regardless of L. monocytogenes infection. (A) Immunoblot analysis of Mic10 levels in HCT116 cells transiently transfected with control plasmid (Ctrl) or a plasmid constitutively expressing C-terminal FLAG-tagged Mic10 (Mic10-FLAG). Both endogenous Mic10 and exogenous Mic10-FLAG were detected with an anti-Mic10 antibody. Beta-actin protein was used as the loading control. (B) Immunofluorescence analysis of HCT116 cells transiently transfected with Ctrl or Mic10-FLAG plasmids which were left uninfected (NI) or infected (MOI 20, 2 h) with GFP-expressing wild-type (WT) or LLO-deficient (Δ*hly*) L. monocytogenes. Mitochondria (anti-Tom20) are shown in red, Mic10-FLAG (anti-FLAG) in green, nuclei (Hoechst 33342) in blue, and bacteria (L. monocytogenes) in yellow. The white box indicates a region of the mitochondrial network magnified (2×) in the inset shown at the bottom (mitochondrial labeling only). Scale bar (top right), 10 μm. (C and D) Quantification of the degree of mitochondrial fragmentation in HCT116 cells analyzed as described for panel B. Mitochondrial network morphology was analyzed using the morphometric ImageJ plugin tool MiNA on mitochondrion-labeled images. Fragmentation degree per cell was determined by the ratio between the number of individual mitochondrial particles and the total mitochondrial area. Scatter plots show mitochondrial fragmentation degree (C) and rod/branch length (D) values for each cell (dots, *n* > 70) and means (horizontal bar) ± SD and are representative of results from three independent experiments. Significance was determined by a Kruskal-Wallis test (ns, not significant; ***, *P < *0.001; ****, *P < *0.0001).

Next, we examined whether increasing Mic10 levels would have an impact on *Listeria*-dependent mitochondrial fission. We followed the experimental approach described earlier but, instead of using siRNA, cells were transfected with a plasmid driving the constitutive expression of a C-terminal FLAG fusion of the human Mic10 protein (Mic10-FLAG) or with the empty parental plasmid as a negative control (Ctrl). We observed that constitutive expression of Mic10-FLAG resulted in a concomitant reduction of the endogenous Mic10 levels (Fig. 3A). This effect was previously reported for both Mic10 and Mic60 and suggests regulation of the total levels of MICOS complex proteins (29). Whereas the Ctrl cells displayed significantly increased mitochondrial network fragmentation upon infection with wild-type bacteria, cells expressing high levels of Mic10-FLAG showed a highly vesiculated mitochondrial network, even in the absence of infection (Fig. 3B to D). Unlike Mic10 depletion, which did not affect mitochondrial network morphology (Fig. 2B to D), constitutive Mic10-FLAG overexpression caused a collapse of the mitochondrial network. To look for a nonspecific effect of the Mic10-associated FLAG tag, we assessed the degree of mitochondrial fragmentation in cells transfected with a plasmid expressing a C-terminal FLAG-tagged variant of the ATP synthase membrane subunit DAPIT, an unrelated small protein also localized in the inner mitochondrial membrane. Unlike the Mic10-FLAG observations, expression of DAPIT-FLAG did not result in increased mitochondrial fragmentation (Fig. S2), ruling out an unanticipated effect of the FLAG tag and highlighting the specificity of Mic10 in this process.

10.1128/mBio.03171-19.2 FIG S2 Increased mitochondrial fragmentation in cells expressing Mic10-FLAG is specific to Mic10. (A) Immunofluorescence analysis of HCT116 cells transiently transfected with control plasmid (Ctrl) or plasmids constitutively expressing C-terminal FLAG-tagged Mic10 (Mic10-FLAG) or DAPIT (DAPIT-FLAG). Mitochondria (anti-Tom20) are shown in red, Mic10-FLAG or DAPIT-FLAG (anti-FLAG) in green, and nuclei (Hoechst 33342) in blue. The white box indicates a region of the mitochondrial network magnified (2×) in the inset shown at the bottom (mitochondrial labeling only). Scale bar (top right), 10 μm. (B and C) Quantification of the degree of mitochondrial fragmentation in HCT116 cells analyzed as described for panel A. Mitochondrial network morphology was analyzed using the morphometric ImageJ plugin tool MiNA on mitochondrion-labeled images. Fragmentation degree per cell was determined by the ratio between the number of individual mitochondrial particles and the total mitochondrial area. Scatter plots show mitochondrial fragmentation degree (B) and rod/branch length (C) values for each cell (dots, *n* > 70) with means (horizontal bar) ± SD and are representative of results from three independent experiments. Significance was determined by a Kruskal-Wallis test (ns, not significant; ****, *P < *0.0001). Download FIG S2, EPS file, 2.1 MB. Copyright © 2020 Carvalho et al. 2020 Carvalho et al. This content is distributed under the terms of the Creative Commons Attribution 4.0 International license.

Overall, these results indicate that L. monocytogenes requires basal Mic10 levels to trigger mitochondrial fission in an LLO-dependent manner. Moreover, together with our proteomic data, they suggest that this mitochondrial network breakdown is a result of increased Mic10 levels in mitochondria.

### Mic10 contributes to efficient L. monocytogenes cellular infection.

**FIG 4:**
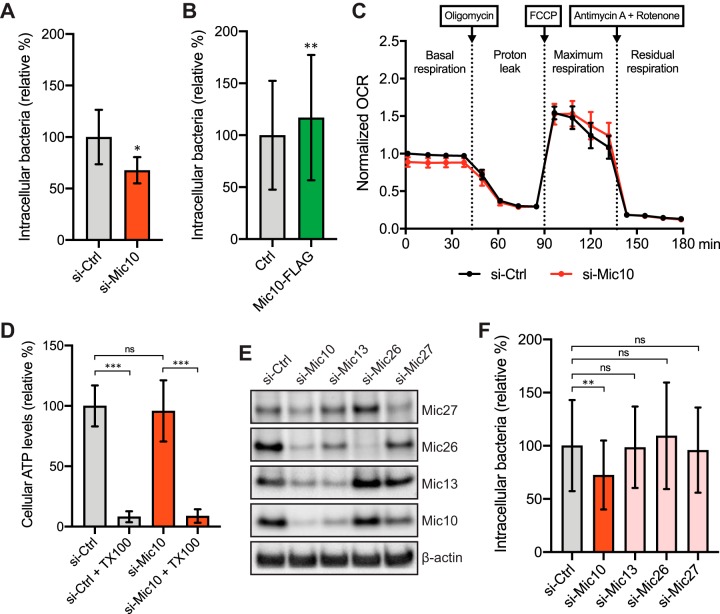
Mic10 contributes to L. monocytogenes cellular infection. (A) Quantification of intracellular bacteria in HCT116 cells transfected with nontargeting control (si-Ctrl) or Mic10-targeting (si-Mic10) siRNAs, following infection with wild-type L. monocytogenes (MOI 20, 2 h). Data represent means ± SD of results from three independent experiments and are expressed as percentages of intracellular bacteria relative to those quantified in si-Ctrl cells. Significance was determined by a paired-ratio test (*, *P < *0.05). (B) Quantification of intracellular bacteria in HCT116 cells transiently transfected with control plasmid (Ctrl) or a plasmid constitutively expressing C-terminal FLAG-tagged Mic10 (Mic10-FLAG), following infection with wild-type L. monocytogenes (MOI 20, 2 h). Data represent means ± SD of results from three independent experiments and are expressed as percentages of intracellular bacteria relative to those quantified in Ctrl cells. Significance was determined by a paired-ratio test (**, *P < *0.01). (C) Oxygen consumption rate (OCR [picomoles per minute]) of HCT116 cells transfected with si-Ctrl or si-Mic10 siRNAs measured in a Seahorse XF analyzer. Electron transport chain inhibitors (oligomycin, FCCP, and antimycin A/rotenone) were added at defined time points to monitor ATP synthesis-associated respiration (proton leak) and maximum and non-mitochondrion-associated (residual) respiratory capacities. Data are expressed as a fraction of the first OCR value (basal respiration) determined for the si-Ctrl cells and are shown as means ± standard errors of the means (SEM) of results from three independent experiments. (D) Cellular ATP levels in HCT116 cells transfected with si-Ctrl or si-Mic10 siRNAs were quantified by luminescence-based plate assay, using an ATPlite luminescence assay kit. Negative controls consist of cells treated with Triton X-100 (TX100). Data are expressed as percentages of ATP levels relative to those quantified in si-Ctrl cells and represent means ± SD of results from three independent experiments. Significance was determined by a matched one-way ANOVA with Sidak’s *post hoc* test (ns, not significant; ***, *P < *0.001). (E) Immunoblot analysis of the levels of Mic10 subcomplex members (Mic10, Mic13, Mic26, and Mic27) in HCT116 cells transfected with si-Ctrl siRNAs targeting Mic10 (si-Mic10), Mic13 (si-Mic13), Mic26 (si-Mic26), or Mic27 (si-Mic27). Beta-actin protein was used as the loading control. (F) Quantification of intracellular bacteria in cells treated as described for panel E after infection with wild-type L. monocytogenes (MOI 20, 2 h). Data represent means ± SD of results from three independent experiments and are expressed as percentages of intracellular bacteria relative to those quantified in si-Ctrl cells. Significance was determined by a matched one-way ANOVA with Dunnett’s *post hoc* test (ns, not significant; **, *P < *0.01).

The dynamic state of the mitochondrial network was reported to play a role in the early steps of L. monocytogenes infection, as cells with fragmented mitochondria were less susceptible to infection, whereas cells with hyperfused mitochondria showed increased infection levels (16). Considering our results regarding the effect of Mic10 levels on mitochondrial morphology, we wondered whether and how Mic10 levels would affect L. monocytogenes infection. We performed gentamicin protection assays in cells depleted of Mic10 or overexpressing Mic10, and, after infection of the cells with wild-type bacteria, we quantified the intracellular bacterial load. The Mic10-depleted cells showed 30% less infection than the control cells (Fig. 4A), whereas cells overexpressing Mic10 showed a 20% increase in levels of intracellular bacteria (Fig. 4B). To determine if the reduced infection levels observed under conditions of Mic10 depletion were due to alterations in cellular bioenergetics caused by defects in mitochondrial function and energy metabolism, we analyzed the mitochondrial respiratory and ATP production capacity. The oxygen consumption rate (OCR) and ATP levels in si-Mic10 cells were similar to those in si-Ctrl cells (Fig. 4C and D), in agreement with other studies (30, 31). These results suggest that the effect of Mic10 on L. monocytogenes infection is not caused by changes in mitochondrial energy production. Furthermore, we show that the lack of mitochondrial fragmentation in infected si-Mic10 cells is not a consequence of the reduced infection efficiency, as wild-type cells challenged with noninvasive bacteria lacking internalins A and B (Δ*inlAB*) (Fig. S3A) displayed mitochondrial fragmentation levels comparable to those seen with wild-type *Listeria*-infected cells (Fig. S3B and C), as well as increased mitochondrial Mic10 levels similar to what we observed in wild-type *Listeria*-infected and LLO-treated cells (Fig. S3D).

10.1128/mBio.03171-19.3 FIG S3 L. monocytogenes-induced mitochondrial fragmentation occurs independently of cell infection. (A) Quantification of intracellular bacteria in HCT116 cells infected with wild-type (WT), LLO-deficient (Δ*hly*), or internalin A and B-deficient (Δ*inlAB*) L. monocytogenes (MOI 20, 2 h). Data represent means ± SD of results from three independent experiments and are expressed as percentages of intracellular bacteria relative to those quantified in L. monocytogenes WT-infected cells. Significance was determined by a matched one-way ANOVA with Dunnett’s *post hoc* test (ns, not significant; **, *P < *0.01). (B and C) Quantification of the degree of mitochondrial fragmentation in HCT116 cells left uninfected (NI) or infected (MOI 20, 2 h) with wild-type (WT), LLO-deficient (Δ*hly*), or internalin A and B-deficient (Δ*inlAB*) L. monocytogenes. Mitochondrial network morphology was analyzed using the morphometric ImageJ plugin tool MiNA on mitochondrion-labeled images. Fragmentation degree per cell was determined by the ratio between the number of individual mitochondrial particles and the total mitochondrial area. Scatter plots show mitochondrial fragmentation degree (B) and rod/branch length (C) values for each cell (dots, *n* > 70) with means (horizontal bar) ± SD and are representative of results from three independent experiments. Significance was determined by a Kruskal-Wallis test (ns, not significant; *, *P < *0.05; ****, *P < *0.0001). (D) Quantification of the mitochondrial fraction of Mic10 in HCT116 cells analyzed as described for panel B. Fluorescence intensities in the entire cell and in mitochondria were measured in Mic10-labeled images using Fiji. Results are expressed as means ± SD (*n* > 99 cells per condition). Significance was determined by a Kruskal-Wallis test (ns, not significant; *, *P < *0.05; **, *P < *0.01; ***, *P < *0.001). Download FIG S3, EPS file, 1.2 MB. Copyright © 2020 Carvalho et al. 2020 Carvalho et al. This content is distributed under the terms of the Creative Commons Attribution 4.0 International license.

Depletion of Mic10 in yeast and mammalian cells results in reduced levels of MICOS complex proteins Mic13, Mic26, and Mic27 (27, 29, 32, 33), which interact closely with Mic10 to form the Mic10 subcomplex (20). We thus assessed if the decreased *Listeria* infection associated with Mic10 knockdown was a consequence of the lower abundance of Mic13, Mic26, and/or Mic27 by performing siRNA-mediated silencing of the corresponding genes in the cells before infection with L. monocytogenes. We confirmed that Mic10 depletion results in decreased levels of the other three members of the Mic10 subcomplex (Fig. 4E). In turn, Mic13 and, to a lesser degree, Mic27 were also found to be necessary to sustain basal Mic10 levels (Fig. 4E), in agreement with previous reports (32, 33). However, quantification of intracellular bacteria revealed no difference between control cells and cells depleted for Mic13 or Mic26 or Mic27, in contrast to the attenuated infection in si-Mic10 cells (Fig. 4F). This result demonstrates that none of these MICOS complex subunits is individually required for *Listeria* cellular infection, supporting the idea of a unique role of Mic10 in this process. In agreement with this finding, besides Mic10, none of the other six subunits of the metazoan MICOS complex (Mic13, Mic19, Mic25, Mic26, Mic27, and Mic60) showed significantly changed levels with L. monocytogenes infection in our proteomic analysis. We then investigated if the increased Mic10 levels resulted from infection-driven transcriptional upregulation. Real-time quantitative PCR (RT-qPCR) analysis of RNAs from cells infected or not with wild-type bacteria showed that the relative levels of transcripts coding for Mic10 or any other MICOS complex subunits were unchanged upon L. monocytogenes infection (Fig. S4). This result suggests that, instead of upregulating Mic10 transcription to elevate its protein levels, L. monocytogenes infection promotes an accumulation of Mic10 protein in mitochondria through an unknown posttranscriptional mechanism.

10.1128/mBio.03171-19.4 FIG S4 Transcription of Mic10 or other MICOS complex genes is not upregulated upon L. monocytogenes infection. Data represent results of analysis of gene expression of human MICOS complex subunits Mic10, Mic13, Mic19, Mic25, Mic26, Mic27, and Mic60 in response to L. monocytogenes infection. HCT116 cells were left uninfected (NI) or infected (MOI 20, 2 h) with wild-type (WT) or LLO-deficient (Δ*hly*) L. monocytogenes, and total cellular RNAs were isolated and used for RT-qPCR analysis. Data represent means ± SD of results from three independent experiments and are presented as fold change in transcript levels relative to those in NI cells. Significance was determined by an ordinary two-way ANOVA with Dunnett’s *post hoc* test (ns, not significant). Download FIG S4, EPS file, 0.4 MB. Copyright © 2020 Carvalho et al. 2020 Carvalho et al. This content is distributed under the terms of the Creative Commons Attribution 4.0 International license.

Overall, these results indicate that *Listeria* cellular infection efficiency is specifically and positively correlated with increased mitochondrial levels of Mic10.

## DISCUSSION

Several intracellular bacterial pathogens, such as L. monocytogenes, Legionella pneumophila, Shigella flexneri, Chlamydia trachomatis, and Mycobacterium tuberculosis, interfere with mitochondrial dynamics to create an intracellular environment suited to their survival and persistence (14–16, 18, 34–36). In particular, L. monocytogenes was previously shown to induce mitochondrial fission early in infection and to cause a metabolic slowdown (16, 18), delaying mitochondrion-dependent cellular responses, such as type III interferon signaling (37). Here, we employed quantitative proteomics to characterize the mitochondrial response to L. monocytogenes infection and to search for host factors involved in L. monocytogenes-induced mitochondrial fission. We revealed the MICOS complex protein Mic10 as a new player in this process. We showed that L. monocytogenes infection increases Mic10 levels specifically in mitochondria in an LLO-dependent manner and that Mic10 abundance is positively correlated with L. monocytogenes-induced mitochondrial fragmentation and host cell infection. This supports a model whereby L. monocytogenes infection promotes elevated mitochondrial Mic10 levels to trigger organellar fission and favor infection.

Our proteomic approach yielded a degree of mitochondrial enrichment (36%) and a level of mitochondrial proteome coverage (53%) comparable to those reported in other studies using different mitochondrial isolation and mass spectrometry protocols (38–40). One of those studies explored the host mitochondrial response to M. tuberculosis infection, showing that virulent strains, in contrast to avirulent bacteria, increased mitochondrial energy production and protected host cells from apoptosis. These changes were partially supported at the protein level, with upregulation of proteins involved in respiration and antiapoptotic mechanisms and reduced levels of proteins linked to antimicrobial responses (38). Our proteomic data also suggest that mitochondrial translation and respiration are enhanced in response to L. monocytogenes infection, possibly to compensate for the drop in mitochondrial membrane potential (16).

Analyzing mitochondrion-associated proteins that were differentially regulated in an LLO-dependent manner, we found that some proteins, such as lanosterol synthase (LSS) and Bcl2-like protein 1/Bcl-xL (BCL2L1), were less abundant in mitochondria of infected cells. LSS was shown to participate in protection against mitochondrial stress induced by 1-methyl-4-phenylpyridinium (MPP^+^) through the production of lanosterol in mitochondria, which can promote uncoupling and clearance of dysfunctional mitochondria through mitophagy (41). It is conceivable that the decrease in mitochondrial levels of LSS affects lanosterol-dependent regulation of mitochondrial maintenance pathways, contributing to destabilization and collapse of the mitochondrial network upon *Listeria* infection. BCL2L1 is an antiapoptotic factor that is also involved in mitochondrial dynamics, as it was shown to promote increased mitochondrial length and biomass (42). Thus, lower mitochondrial BCL2L1 levels could also contribute to the increased mitochondrial fragmentation observed as a result of *Listeria* infection. Other proteins, such as Mic10, showed an LLO-dependent rise in their mitochondrial levels with infection. In this study, we chose to explore Mic10 because it represents a bona fide IMM protein with well-characterized membrane-shaping properties (27, 28, 43–45) that could play an unsuspected role in mitochondrial fission. This choice was also supported by recent evidence pointing to the possibility of mitochondrial fission starting with constriction of the IMM, independently of the OMM (46). This process is driven functionally by intramitochondrial Ca^2+^ influx and spatially by ER-mitochondrion contact sites, suggesting a link with LLO-mediated Ca^2+^ flux dynamics and attesting the ER involvement, as we previously reported (16, 18). Importantly, the MICOS complex is a featured player in this mechanism (46, 47), although existing evidence has not shown the involvement of Mic10 in this process. We hypothesized that elevated mitochondrial Mic10 levels could drive deregulated IMM remodeling, resulting in mitochondrial fission. In support of this assumption, we showed that L. monocytogenes does not increase mitochondrial fragmentation in Mic10-depleted cells. In contrast, we observed clear fragmentation in cells overexpressing Mic10, even in the absence of bacteria. Others have reported that high Mic10 levels disrupt crista structure (27) and that Mic10 knockdown or knockout also results in absent crista junctions and detached cristae that stack in the matrix (27–31, 43, 48). These phenotypes attest the importance of Mic10 in IMM structure maintenance and suggest that L. monocytogenes could target Mic10 to induce IMM remodeling and trigger mitochondrial fission. We did not observe changes of the mitochondrial morphology in Mic10-depleted cells, implying that the ultrastructural defects caused by Mic10 knockdown are not sufficient to elicit mitochondrial fragmentation, in contrast to the disruptive effect of excessive Mic10 levels. Consistently, knockdown of other MICOS subunits, such as Mic60 (29), Mic19, and Mic25 (49), did not cause mitochondrial fragmentation, although Mic60- and Mic19-depleted mitochondria did show bulb-like enlargements (29, 49). Similar features were reported in Mic10-null yeast mitochondria (50), but we did not observe them in our si-Mic10 cells.

Surprisingly, the enrichment of Mic10 in mitochondria upon L. monocytogenes infection was not due to increased Mic10 transcription, which suggests that Mic10 accumulation in mitochondria occurs at the posttranscriptional level. This could have resulted from increased import or reduced turnover of Mic10 in mitochondria. As a nuclear gene-encoded protein, Mic10 is imported from the cytosol via the mitochondrial protein import machinery (51, 52). However, as other MICOS proteins are similarly imported (52) and as our proteomics data showed no significant changes in their mitochondrial levels, it seems unlikely that increased Mic10 levels are caused by enhanced Mic10-specific mitochondrial import. Protein turnover in mitochondria is carried out by proteases residing in the different subcompartments (53). Interestingly, two of these proteases, Yme1L and Oma1, were reported to participate in the processing of Mic60 and Mic19, respectively (29, 49), suggesting that they may participate in Mic10 proteolysis. Further knockdown or loss-of-function experiments should clarify the involvement of Yme1L and/or Oma1 in Mic10 turnover.

Cells with fragmented mitochondria were shown to be less extensively infected by L. monocytogenes, raising the hypothesis that prefragmented mitochondria are more resistant to the L. monocytogenes-induced bioenergetic slowdown (16). Here, we demonstrated that L. monocytogenes infection is partially impaired in cells with reduced Mic10 abundance, suggesting that Mic10-dependent mitochondrial fission induced by L. monocytogenes is important for subsequent cellular infection. In contrast, bacterial infection was improved in cells transfected with DNA driving Mic10 overexpression. Since not every cell overexpressed Mic10, it is possible that this increase may be higher. Further experiments using stable clones of Mic10-overexpressing cells should be helpful to confirm that *Listeria* infection is enhanced due to increased mitochondrial Mic10 levels.

An important issue is how L. monocytogenes manipulates events taking place inside mitochondria, even at early steps of infection, when it is entering cells or possibly still in the extracellular medium. LLO secreted by L. monocytogenes is a main trigger, mediating Ca^2+^ influx into the host cytoplasm (16, 54, 55). Mitochondria take up Ca^2+^ from the cytosol, or from the ER at MAMs (mitochondrion-associated ER membranes), via the mitochondrial calcium uniporter (MCU) complex (3), which includes mitochondrial calcium uptake protein 1 (MICU1). This MCU complex component controls Ca^2+^ crossing the MCU channel and thereby the intramitochondrial Ca^2+^ concentration (56). Interestingly, MICU1 was identified in our proteomic analysis, showing an apparent enrichment with L. monocytogenes infection in an LLO-dependent manner (see Table S1 in the supplemental material). Alternatively, mitochondrial Ca^2+^ efflux is mediated by, among others, the sodium/calcium exchanger NCLX (57), which can be activated by protein kinase A (PKA)-mediated phosphorylation (58). The catalytic subunit alpha of PKA (PRKACA) is one of four mitochondrion-related proteins that are less abundant with infection in an LLO-dependent manner, suggesting that PKA-mediated NCLX activation is impaired during L. monocytogenes infection. MICU1 upregulation and NCLX inhibition could result in increased intramitochondrial Ca^2+^ concentrations and, among other effects, in a generalized collapse of the mitochondrial network (59). An investigation of the contribution of these mitochondrial proteins could clarify a role for mitochondrial Ca^2+^ uptake in L. monocytogenes-dependent and possibly also Mic10-dependent mitochondrial fragmentation.

In conclusion, this work represents the first proteomic analysis of the mitochondrial response to L. monocytogenes infection and revealed a novel actor in mitochondrial dynamics that is specifically manipulated by L. monocytogenes to create the ideal setting for host cell infection.

## MATERIALS AND METHODS

### Bacterial strains, cell lines, and growth conditions.

The following Listeria monocytogenes strains were used in this study: wild-type EGD (BUG 600), its isogenic LLO mutant EGDΔ*hly* (BUG 3650), and green fluorescent protein (GFP)-expressing strains EGD-cGFP (BUG 2539), EGDΔ*hly*-cGFP (BUG 2786), and EGDΔ*inlAB*-cGFP (BUG 2777). Bacteria were grown at 37°C in brain heart infusion (BHI) media (Difco, BD) supplemented, when required, with chloramphenicol (7 μg/ml). The following tissue culture cell lines were used in this study: HCT116 (human colorectal adenocarcinoma; ATCC CCL-247) and U2OS (human osteosarcoma; ATCC HTB-96). Cells were maintained in McCoy’s 5A GlutaMAX medium (Gibco), supplemented with 1 mM nonessential amino acids (Gibco) and 10% (vol/vol) fetal bovine serum (FBS) (BioWest), and grown at 37°C in a humidified 10% CO_2_ atmosphere.

### Cell transfection.

For transient gene knockdown, cells were reverse transfected with siRNAs in 24-well plates, using Lipofectamine RNAiMax (Invitrogen) according to the manufacturer’s instructions, except that McCoy’s 5A was used as the dilution medium. The medium was changed the following day, and the cells were assayed 48 h posttransfection. siRNA duplexes were used at the following concentrations: siRNA Universal Negative Control #1 (Sigma-Aldrich) and Mic10 (5′-CGGAUGCGGUCGUGAAGAUTT-3′; Eurofins Genomics) at 100 nM; Mic13 (Ambion, Silencer Select catalog no. s195661), Mic26 (Ambion, Silencer Select catalog no. s35601), and Mic27 (Ambion, Silencer Select catalog no. s225655) at 20 nM. For transient overexpression, cells were seeded in 24-well plates 1 day before transfection with 0.5 μg of plasmid DNA, using jetPRIME (Polyplus Transfection) according to the manufacturer’s instructions. Cells were assayed 24 h posttransfection. The expression plasmids pcDNA3.1(+)-MINOS1-DYK (GenScript, ORF cDNA clone OHu15514) and pCMV6-ATP5MD-Myc-DDK (OriGene, TrueORF Gold clone RC203316) were used to express FLAG-tagged Mic10 and DAPIT, respectively. Negative-control cells were transfected with pcDNA3.1(+) (Invitrogen). For immunofluorescence, cells were seeded in wells containing glass coverslips.

### Cell infections.

For cell infection assays, confluent monolayers were incubated for 1 h at 37°C in FBS-free cell culture medium alone (noninfected cells) or inoculated with logarithmic-phase bacteria (optical density at 600 nm [OD_600_] of 0.6 to 1.0) at a multiplicity of infection (MOI; bacteria/cell) of 20 (for HCT116 cells) or 50 (for U2OS cells). The medium was removed, and the cells were incubated for another hour (total infection time, 2 h) at 37°C in FBS-containing culture medium supplemented with 20 μg/ml gentamicin sulfate (Sigma-Aldrich), to kill extracellular bacteria. Cells were then washed with Dulbecco’s phosphate-buffered saline (DPBS; Gibco) before processing for further analyses was performed. For immunofluorescence assays, cells were infected with GFP-expressing L. monocytogenes strains. To quantify intracellular bacterial levels, infected cells were lysed in ice-cold 0.2% (vol/vol) Triton X-100–DPBS, serially diluted in DPBS, and plated on BHI agar plates. CFU counts were performed after 24 h of incubation at 37°C, and bacterial numbers were normalized to the inoculum concentration.

### Western blotting.

For assessment of gene knockdown or overexpression, protein levels were analyzed by Western blotting. Cells were lysed in the well with 2× NuPAGE LDS sample buffer (Invitrogen), and lysates were resolved by SDS-PAGE in NuPAGE 4% to 12% Bis-Tris gels (Invitrogen) using MES (morpholineethanesulfonic acid) SDS running buffer (Invitrogen). Proteins were blotted onto a polyvinylidene difluoride (PVDF) membrane (iBlot gel transfer stacks; Invitrogen) using an iBlot gel transfer device (Invitrogen). Immunoblotting was performed overnight at 4°C with the following primary antibodies: rabbit polyclonal anti-C1ORF151/Mic10 (Abcam; ab84969) (1:500), rabbit polyclonal anti-C19orf70/Mic13 (Proteintech) (1:1,000), rabbit polyclonal anti-ApoO/Mic26 (Atlas Antibodies) (1:500), mouse monoclonal anti-ApoOL/Mic27 clone G-6 (Santa Cruz Biotechnology) (1:250), and mouse monoclonal anti-β-actin clone AC-15 (Sigma-Aldrich) (1:10,000). After incubation with horseradish peroxidase (HRP)-conjugated anti-mouse or anti-rabbit secondary antibodies (Abliance), membranes were incubated with ECL Prime (Amersham) and protein signals were acquired in a ChemiDoc XRS+ system (Bio-Rad Laboratories).

### Mitochondrial isolation and LC-MS/MS sample preparation.

For label-free quantitative proteomic analysis of mitochondria, HCT116 cells (∼5 × 10^7^) cultivated in 150-mm-diameter dishes were left uninfected (NI) or infected either with wild-type (WT) or LLO-deficient (Δ*hly* mutant) strain EGD, as described above. Three independent biological replicates were prepared and analyzed for each condition. After infection, mitochondria were isolated from cells by magnetic immunoaffinity separation, using a mitochondrion isolation kit (human; Miltenyi Biotec). For this, cells were washed with ice-cold DPBS and scraped in ice-cold kit lysis buffer (1 ml/10^7^ cells) containing a cocktail of protease inhibitors (cOmplete, EDTA-free; Roche). Cells were then lysed in a Potter-Elvehjem homogenizer (∼50 strokes), with lysis monitored by trypan blue staining. Lysates were centrifuged for 5 min at 800 × *g* (4°C) to pellet unbroken cells, and the supernatant was recovered for magnetic labeling and separation of mitochondria as detailed in the kit instructions. Purified mitochondria were resuspended in urea lysis buffer (20 mM HEPES [pH 8.0], 8 M urea), and protein concentrations were measured with a bicinchoninic acid (BCA) protein assay kit (Pierce). Proteins were reduced for 30 min at 55°C in the presence of 25 mM dithiothreitol (DTT) and were then alkylated for 15 min in the dark in the presence of 50 mM iodoacetamide. Samples were diluted 2-fold with 20 mM HEPES (pH 8.0), and proteins were digested with Lys-C (Promega) at a protease/protein ratio of 1:100 (wt/wt) for 4 h at 37°C. Samples were again diluted 2-fold and incubated overnight at 37°C with trypsin (sequencing-grade modified; Promega) at a 1:50 (wt/wt) ratio. Formic acid (FA) was added at 1% (vol/vol), and after 10 min on ice, samples were centrifuged for 10 min at 10,000 × *g* to pellet any insoluble material. Peptides in the supernatant were purified in Sep-Pak C_18_ cartridges (Waters), lyophilized, dissolved in solvent A (0.1% [vol/vol] FA, water–acetonitrile [ACN] [98:2 {vol/vol}]) and quantified by absorbance at 280 nm (NanoDrop; Thermo Fisher Scientific) prior to analysis by LC-MS/MS.

### LC-MS/MS and data analysis.

LC-MS/MS analysis of purified peptides (∼2 μg) was conducted in an EASY-nLC 1000 system (Thermo Scientific) coupled to an Orbitrap Q Exactive Plus mass spectrometer (Thermo Scientific). Peptides were loaded at 250 nl/min on a home-made analytical column (Dr. Maisch GmbH) (50 cm by 75 μm; bead size, 1.9 μm; Reprosil-Pur Basic C_18_ beads) and eluted with a nonlinear gradient of solvent B (0.1% [vol/vol] FA, water/ACN [2:8 {vol/vol}]) from 3% to 7% for 7 min, 7% to 34% for 100 min, 34% to 62% for 40 min, and 62% to 75% for 10 min. The full run time, including column wash and regeneration (100% and 2% solvent B, respectively), each performed for 15 min, was 185 min. The mass spectrometer was operated in data-dependent mode, changing automatically between MS and MS/MS scans for the top 10 most abundant ion species. Full-scan MS spectra (scan range, 300 to 1,700 *m*/*z*) were acquired at a resolution of 70,000 (*m*/*z* 400), with a target value of 3 × 10^6^ ions and a maximum injection time of 20 ms. The 10 most intense precursor ions above an intensity threshold value of 1.7 × 10^5^ and with charge states between +2 and +6 were isolated (1.6 Th window; dynamic exclusion of 45 s) for HCD fragmentation at 28% normalized collision energy, after reaching a target value of 1 × 10^6^ in a maximum of 60 ms. MS/MS spectra (scan range, 200 to 2,000 *m*/*z*) were acquired at a resolution of 17,500 (at *m*/*z* 400). Spectra were searched against a database of 20,198 human proteins (UniProtKB/Swiss-Prot, released on 12 April 2017) using MaxQuant software (version 1.5.8.3) (60) with its Andromeda search engine (61). Trypsin was selected as a protease, allowing cleavage before proline residues and up to three missed cleavages. Mass tolerance levels for precursor and fragment ions were set to 5 ppm and 20 ppm, respectively; the maximum peptide charge was set to +7, and the minimum peptide length was set to seven amino acids. Carbamidomethylation of cysteines was set as a fixed modification, while oxidation of methionines and acetylation of protein N termini were set as variable modifications. A false-discovery rate (FDR) of 1% was set at both the protein and peptide levels. A match-between-runs feature was enabled between biological replicates with a matching time window of 1 min and an alignment time window of 20 min. Only proteins identified by at least one unique or razor peptide were retained, and a minimum ratio count of two unique/razor peptides was necessary for quantification via the MaxLFQ algorithm (62). MaxQuant output data (ProteinGroups.txt file) were further analyzed using Perseus software (version 1.6.1.2) (63). Proteins identified as contaminants, identified only by site, or identified as reverse database hits were filtered out, and label-free quantification (LFQ) intensity values were log2 transformed. Proteins not detected in all three biological replicates under at least one condition were removed, and missing values were imputed from a normal distribution around the detection limit. A multiple-sample test (analysis of variance [ANOVA]) was performed (FDR = 0.05, artificial within-group variance [S_0_] = 1) to determine which proteins showed a statistically significant (>2-fold) change in level across all conditions. Statistically significant ANOVA hits were then subjected to Tukey’s *post hoc* testing (FDR = 0.05) to identify the conditions under which there were statistically significant protein level changes. Statistically overrepresented Gene Ontology (GO) biological process terms were determined using the PANTHER enrichment analysis tool (http://www.pantherdb.org/; 2 February 2019 release version) (64). The Integrated Mitochondrial Protein Index (IMPI) reference mitochondrial gene list (version Q2, June 2018 release version) integrated in MitoMiner (http://mitominer.mrc-mbu.cam.ac.uk/) (65) was used to classify identified proteins as mitochondrial. A heat map with hierarchical clustering (Euclidean distance; average linkage) of Z-score-normalized protein intensity levels was generated using the Heatmapper Web server (http://www.heatmapper.ca/) (66).

### Immunofluorescence.

Cells grown on glass coverslips were fixed for 15 min at room temperature in 4% (vol/vol) paraformaldehyde–PBS, permeabilized for 5 min in 0.5% (vol/vol) Triton X-100–PBS, and blocked for 20 min in blocking buffer (1% [wt/vol] bovine serum albumin [BSA], 10% [vol/vol] goat serum–PBS). Labeling with primary and fluorophore-conjugated secondary antibodies or dyes was performed in blocking buffer for 1 h at room temperature. Cells were washed three times in PBS between steps after fixation, except after blocking. Coverslips were mounted onto microscope slides with FluoroMount-G mounting medium (Interchim) and imaged the next day or stored in the dark at 4°C. The primary antibodies were used as follows: rabbit polyclonal anti-C1ORF151/Mic10 (Abcam, ab84969) (1:200), mouse monoclonal anti-Tom20 clone 29 (BD Transduction Laboratories) (1:200), rabbit polyclonal anti-Tom20 clone F-10 (Santa Cruz Biotechnology) (1:200), and mouse monoclonal anti-FLAG clone M2 (Sigma-Aldrich) (1:100). Anti-rabbit and anti-mouse antibodies conjugated to Alexa Fluor 568 and Alexa Fluor 647 dyes (Molecular Probes) (1:500) were used as secondary antibodies; Hoechst 33342 (Molecular Probes) was used to stain DNA. Cells were analyzed in a Zeiss AxioObserver.Z1 inverted microscope (Carl Zeiss AG) equipped with a high-speed CSU-X1 spinning-disk confocal system (Yokogawa) and an Evolve electron-multiplying charge-coupled-device (EM-CCD) camera (Photometrics). Single-focal-plane images were acquired through a Plan-Apochromat 63×/1.4 Ph3 oil objective across multiple wavelength channels, using MetaMorph software (version 7.7.9.0). Fiji was used for image processing, including channel color selection, brightness and contrast adjustment, addition of scale bars, and generation of composite images.

### Mitochondrial morphology analysis.

Confocal images of cells were taken from various fields of view randomly selected across the entire coverslip area, and their mitochondrial morphology was analyzed using the semiautomated morphometric tool MiNA within Fiji (67). Mitochondrial networks (labeled with anti-Tom20) from individual cells were selected and digitally isolated before batch analysis. From the output data, we determined the mitochondrial fragmentation status per cell by using the values listed under “Individuals” (number of unbranched mitochondrial particles, e.g., puncta and rods), normalized to “Mitochondrial footprint” (mitochondrial area), as well as those under “Mean Rod/Branch Length.” A minimum of 50 cells were analyzed per condition, in a total of three independent experiments.

### Cellular respiration and ATP assays.

Cellular respiration was evaluated by measurement of the oxygen consumption rate (OCR) using a Seahorse XFe96 analyzer (Agilent Technologies). Cells were seeded 48 h before the assay in a Seahorse XF96 cell culture microplate (Agilent Technologies) at a density of 1.5 × 10^4^ cells/well (six wells per condition). One hour before the assay, cell medium in the microplate was replaced with prewarmed Seahorse XF base medium (Agilent Technologies) supplemented with 3 g/liter glucose, 1.5 mM l-glutamine, and 1 mM pyruvate (Gibco) (adjusted to pH 7.4). Cells were placed again at 37°C in a non-CO_2_ incubator until the start of the assay. In the meantime, the injection ports of a precalibrated Seahorse XFe96 sensor cartridge (Agilent Technologies) were loaded with stock solutions of (i) ATP synthase inhibitor oligomycin A (Sigma-Aldrich), (ii) uncoupler carbonyl cyanide-4-(trifluoromethoxy)phenylhydrazone (FCCP; Sigma-Aldrich), and (iii) complex I and complex III inhibitors rotenone and antimycin A (Sigma-Aldrich). Each drug was injected into each well at a final concentration of 1 μM to measure basal, proton leak, and maximum and residual respiration. The assay method consisted of an initial cycle of four OCR measurements, which were repeated after every drug injection, for a total run of ∼180 min. Data were analyzed using Wave software (Agilent Technologies). To compare results from different experiments (*n* = 3), OCR values were normalized to that measured at the first time point of the control group (set at 1). To measure cellular ATP levels, a luminescence-based assay was performed using an ATPlite kit (Perkin-Elmer) according to the manufacturer’s instructions. Cells were seeded in a white 96-well plate, and luminescence was recorded in a Cytation 5 microplate reader (Biotek).

### Total RNA extraction and RT-qPCR.

For analysis of gene expression, real-time quantitative PCR (RT-qPCR) was performed on RNAs from HCT116 cells grown in 6-well dishes and infected or not with L. monocytogenes (see above). Total RNAs were extracted from cells using an RNeasy Mini Kit (Qiagen) according to the manufacturer’s instructions. After infection, cells (∼3 × 10^6^) were washed with DPBS and directly lysed by addition of 350 μl of lysis buffer supplemented with 1% (vol/vol) β-mercaptoethanol, followed by ∼10 passages through a 23-gauge needle. RNAs were treated for genomic DNA (gDNA) contamination by performing an on-column DNase digestion protocol, as described in the kit, and were eluted in 50 μl nuclease-free water. RNA concentration and purity were determined using a NanoDrop instrument (Thermo Fisher Scientific), and RNA integrity was assessed by agarose gel electrophoresis. Purified RNAs (1 μg) were reverse transcribed to cDNA with a blend of oligo(dT) and random primers, using a QuantiTect reverse transcription kit (Qiagen) according to the manufacturer’s instructions. RT-qPCR was performed in 10-μL reaction mixtures containing 0.2 μl cDNA, 5 μl SsoFast EvaGreen Supermix (Bio-Rad Laboratories) (2×), and 0.4 μM forward and reverse primers (Eurofins Genomics) (see Table S2 in the supplemental material). Amplification reactions were conducted in a CFX384 real-time PCR system (Bio-Rad Laboratories) with the following cycling protocol: 1 cycle of 95°C (30 s) and 40 cycles of 95°C (5 s) and 60°C (5 s). For each condition, each target gene was analyzed in triplicate and no-template (water) and gDNA contamination controls (unconverted RNA) were included for each primer pair. Data were analyzed by the comparative threshold cycle (ΔΔ*C_T_*) method after normalization of target gene values, in both test and control samples, to those of a housekeeping gene (human β-actin gene).

10.1128/mBio.03171-19.6 TABLE S2 Primers used for analysis of MICOS complex gene expression by real-time quantitative PCR. Download Table S2, PDF file, 0.01 MB. Copyright © 2020 Carvalho et al. 2020 Carvalho et al. This content is distributed under the terms of the Creative Commons Attribution 4.0 International license.

### Statistics.

Statistical analyses were performed in Prism 8 (GraphPad Software). Differences between group means were considered statistically significant at a *P* value of <0.05. Significance levels are indicated as follows: ns, not significant (*P* = >0.05); *, *P* = <0.05; **, *P* = <0.01; ***, *P* = <0.001, ****, *P* = <0.0001.

### Data availability.

Mass spectrometry proteomics data have been deposited in the ProteomeXchange Consortium via the PRIDE (68) partner repository with the data set identifier PXD014667.
