# Supplementary material for: Listeria monocytogenes Exploits Mitochondrial Contact Site and Cristae Organizing System Complex Subunit Mic10 To Promote Mitochondrial Fragmentation and Cellular Infection
Source: mBio. 2020 Feb 4;11(1):e03171-19. doi: 10.1128/mBio.03171-19 (PMC7002346; doi:10.1128/mBio.03171-19)
Supplement: TABLE S1 [file mBio.03171-19-st001.pdf]

Supplementary Table S1 – List of the 35 differentially abundant proteins identified by quantitative label-free shotgun proteomics and annotated as "mitochondrial" according to IMPI database. Proteins with significantly changed levels were determined by ANOVA (FDR=0.05, S0=1) and pairs of significantly different conditions were determined by Tukey's post hoc test (FDR=0.05). Proteins are ordered by ANOVA significance level (q-value).

UniProt acession number	Gene name	Protein name	Significant change in protein levels				Unique peptides	Sequence coverage (%)	ANOVA q-value
			NI < WT	NI > WT	WT > Δ*hly*	WT < Δ*hly*			
P31939	ATIC	Bifunctional purine biosynthesis protein PURH; Phosphoribosylaminoimidazolecarboxamide formyltransferase/IMP cyclohydrolase			+		10	24,3	0
Q9UL15	BAG5	BAG family molecular chaperone regulator 5	+		+		3	8,9	0
P0C7P0	CISD3	CDGSH iron-sulfur domain-containing protein 3, mitochondrial			+		4	32,3	0
P24311	COX7B	Cytochrome c oxidase subunit 7B, mitochondrial	+				2	10	0
Q5TGZ0	MINOS1	MICOS complex subunit MIC10	+		+		3	61,5	0
O95178	NDUFB2	NADH dehydrogenase [ubiquinone] 1 beta subcomplex subunit 2, mitochondrial	+		+		3	19	0
P86397	HTD2	Hydroxyacyl-thioester dehydratase type 2, mitochondrial			+		2	29,8	0
Q8N357	SLC35F6	Solute carrier family 35 member F6			+		1	3,2	0
Q9BSF4	TIMM29	Mitochondrial import inner membrane translocase subunit Tim29			+		4	26,9	0
Q9H4I3	TRABD	TraB domain-containing protein			+		4	14,4	0
Q6UW78	UQCC3	Ubiquinol-cytochrome-c reductase complex assembly factor 3			+		1	23,7	0
Q9Y6G3	MRPL42	39S ribosomal protein L42, mitochondrial	+		+		4	36,6	0,0004
P48449	LSS	Lanosterol synthase		+		+	10	20,2	0,0006
Q07817	BCL2L1	Bcl-2-like protein 1		+		+	5	45,5	0,0011
Q5VT66	MARC1	Mitochondrial amidoxime-reducing component 1	+		+		4	18,1	0,0011
O14734	ACOT8	Acyl-coenzyme A thioesterase 8		+			4	20,1	0,0019
Q9NW81	DMAC2	Distal membrane arm assembly complex 2			+		5	26,5	0,0020
Q9HA77	CARS2	Probable cysteine--tRNA ligase, mitochondrial			+		4	9,4	0,0022
Q96BP2	CHCHD1	Coiled-coil-helix-coiled-coil-helix domain-containing protein 1					3	25,4	0,0025
P56378	ATP5MPL	ATP synthase membrane subunit 6.8PL		+			2	32,8	0,0039
P17612	PRKACA	cAMP-dependent protein kinase catalytic subunit alpha		+		+	5	13,7	0,0046
Q9BPX6	MICU1	Calcium uptake protein 1, mitochondrial			+		3	11,8	0,0048
Q8IVP5	FUNDC1	FUN14 domain-containing protein 1	+				1	11	0,0059
Q5BJH7	YIF1B	Protein YIF1B			+		2	12,4	0,0061
Q9H3H1	TRIT1	tRNA dimethylallyltransferase, mitochondrial	+				2	8,4	0,0062
Q92876	KLK6	Kallikrein-6		+			3	21,3	0,0089
Q9UL12	SARDH	Sarcosine dehydrogenase, mitochondrial	+				2	6,8	0,0157
Q9Y230	RUVBL2	RuvB-like 2	+				16	45,6	0,0182
Q96H55	MYO19	Unconventional myosin-XIX	+				5	8,5	0,0237
Q9Y5Z9	UBIAD1	UbiA prenyltransferase domain-containing protein 1		+			3	16,9	0,0257
Q9NVA1	UQCC1	Ubiquinol-cytochrome-c reductase complex assembly factor 1	+				3	12,4	0,0257
Q96GW9	MARS2	Methionine--tRNA ligase, mitochondrial	+		+		8	26,6	0,0338
P30041	PRDX6	Peroxiredoxin-6		+		+	10	56,7	0,0345
O14874	BCKDK	[3-methyl-2-oxobutanoate dehydrogenase [lipoamide]] kinase, mitochondrial			+		4	14,1	0,0374
P09669	COX6C	Cytochrome c oxidase subunit 6C	+		+		7	44	0,0454
