# Supplementary figures and images for: Listeria monocytogenes Exploits Mitochondrial Contact Site and Cristae Organizing System Complex Subunit Mic10 To Promote Mitochondrial Fragmentation and Cellular Infection
Source: mBio. 2020 Feb 4;11(1):e03171-19. doi: 10.1128/mBio.03171-19 (PMC7002346; doi:10.1128/mBio.03171-19)

**A**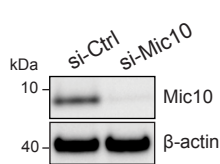**C**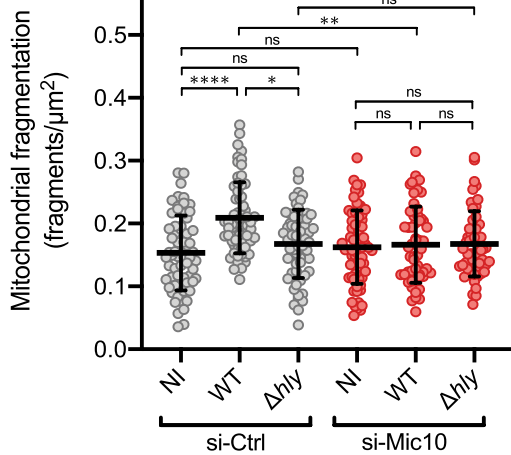**D**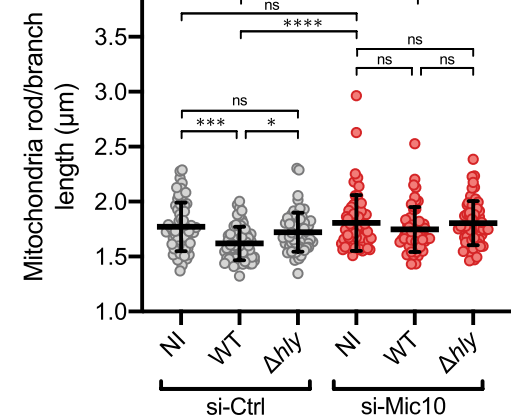**E**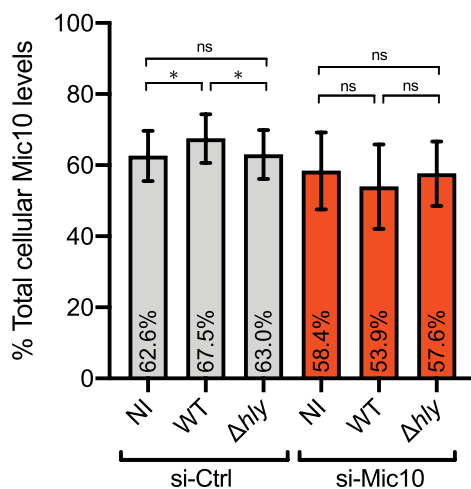**B**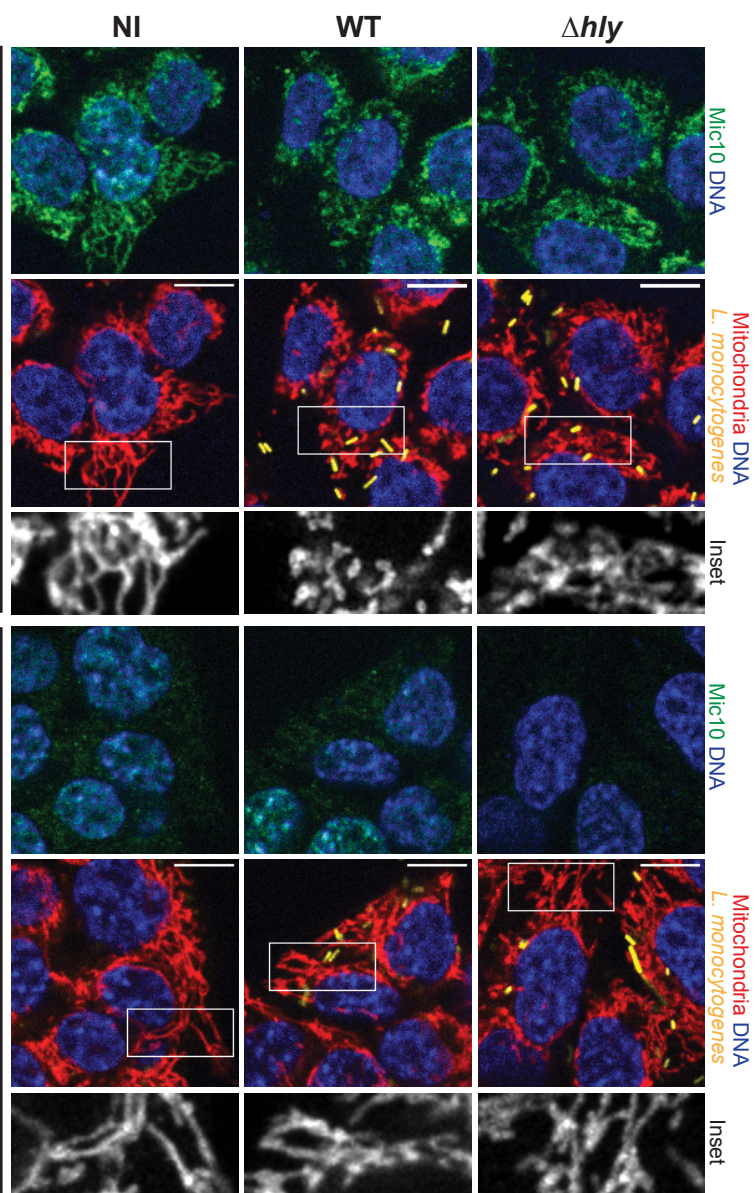**F**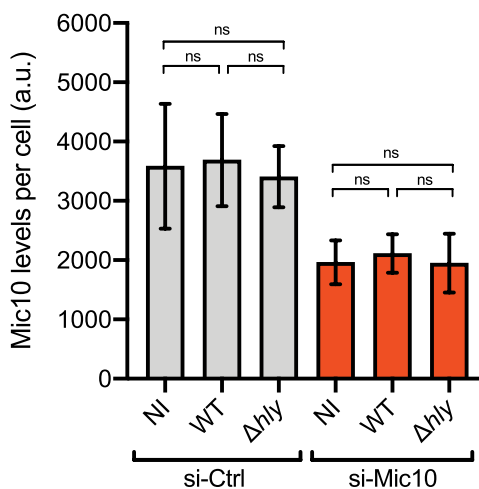

Supplement: FIG S1 [file mBio.03171-19-sf001.pdf]
