# Supplementary material for: Listeria monocytogenes Exploits Mitochondrial Contact Site and Cristae Organizing System Complex Subunit Mic10 To Promote Mitochondrial Fragmentation and Cellular Infection
Source: mBio. 2020 Feb 4;11(1):e03171-19. doi: 10.1128/mBio.03171-19 (PMC7002346; doi:10.1128/mBio.03171-19)
Supplement: TABLE S2 [file mBio.03171-19-st002.pdf]

Supplementary Table S2 – Primers used for analysis of MICOS complex gene expression by real-time quantitative PCR.

Primer name	Sequence (5' to 3')
qPCR-Mic10-Fw	GATGCGGTCGTGAAGATAGG
qPCR-Mic10-Rv	GGAAATCATGCTGACAGTTGG
qPCR-Mic60-Fw	GCAGATACTCTACTTCAGGCAG
qPCR-Mic60-Rv	TGGTTTTCTCTACACTTTCCCG
qPCR-Mic13-Fw	CCAGCCCCTCCAAAGATTTAC
qPCR-Mic13-Rv	CGCCTTCACATACTCCCAG
qPCR-Mic19-Fw	GGACGAGAATGAGAACATCACC
qPCR-Mic19-Rv	ACCATAAGCACCAGAATACCG
qPCR-Mic25-Fw	CTGGAGCGTATTGAGAGGAAG
qPCR-Mic25-Rv	GTAGCAGTGGAGAATCTGGG
qPCR-Mic26-Fw	CCTGAGGGTCAATCGAAGTATG
qPCR-Mic26-Rv	TTGGGCTTAGTTTGGGAGTAC
qPCR-Mic27-Fw	GGGAAAACTGACAACCATGC
qPCR-Mic27-Rv	CATATTTAGACTGGAGCGGTGG
qPCR-ACTB-Fw	GCAGGAGTATGACGAGTCCG
qPCR-ACTB-Rv	AACAACGCATCTCATATTTGGAA
